# Advice from artificial intelligence: a review and practical implications

**DOI:** 10.3389/fpsyg.2024.1390182

**Published:** 2024-10-08

**Authors:** Julia I. Baines, Reeshad S. Dalal, Lida P. Ponce, Ho-Chun Tsai

**Affiliations:** ^1^Department of Psychology, George Mason University, Fairfax, VA, United States; ^2^Department of Psychology, Illinois Institute of Technology, Chicago, IL, United States

**Keywords:** artificial intelligence, algorithm, chatbot, advice, advisor, robo-advisor, virtual assistant, anthropomorphize

## Abstract

Despite considerable behavioral and organizational research on advice from human advisors, and despite the increasing study of artificial intelligence (AI) in organizational research, workplace-related applications, and popular discourse, an interdisciplinary review of advice from AI (vs. human) advisors has yet to be undertaken. We argue that the increasing adoption of AI to augment human decision-making would benefit from a framework that can characterize such interactions. Thus, the current research invokes judgment and decision-making research on advice from human advisors and uses a conceptual “fit”-based model to: (1) summarize how the characteristics of the AI advisor, human decision-maker, and advice environment influence advice exchanges and outcomes (including informed speculation about the durability of such findings in light of rapid advances in AI technology), (2) delineate future research directions (along with specific predictions), and (3) provide practical implications involving the use of AI advice by human decision-makers in applied settings.

## 1 Introduction

Artificial intelligence would…understand exactly what you wanted, and it would give you the right thing…. It would be able to answer any question (Page, 2000).

Recent developments in artificial intelligence (AI) have allowed AI advisors to be incorporated into decision contexts that previously relied solely upon human judgment (Jussupow et al., 2020; Keding and Meissner, 2021). Applications of AI advisors occur in communication, analytics and customer service; manufacturing; infrastructure and agriculture; medical diagnostics and treatment plans; security and emergency responses; and financial advising, among others (Walsh et al., 2019; Metzler et al., 2022; Pezzo et al., 2022; Vrontis et al., 2022). Moreover, recent developments in AI such as ChatGPT and Bard have gripped the popular imagination and shaped public discourse (Roose, 2022; Nellis and Dastin, 2023; Shankland, 2023).

In the current paper, we adopt (Walsh et al.’s, 2019, p. 14) definition of AI as *“a collection of interrelated technologies used to solve problems and perform tasks that, when humans do them, requires thinking.”* Along these lines, we use the term AI as an umbrella term for such technologies; thus, in the current paper, the term “AI” may include artificial intelligence systems, algorithms, conversational agents such as chatbots and social robots, decision support systems, and so forth. For a full list of related terms, see Table 1. Despite this breadth of technologies, our focus is on AI that offers advice to a human decision-maker in the context of an upcoming decision (in a domain such as finance, medicine, security, analytics, employee recruitment and selection, etc.). We therefore use the terminology “AI advice/advisor” and “human advice/advisor” to describe when advice to the human decision-maker comes from an AI versus a human advisor, respectively.^1^

**Table 1:** Advisory technology terms and definitions.

Term	Definition
Algorithm	Script for mathematical calculations; a series of mathematical calculations (Logg, 2017; Logg et al., 2019)
Artificial intelligence (AI)	“A collection of interrelated technologies used to solve problems and perform tasks that, when humans do them, require cognition” (Walsh et al., 2019, p. 14). AI encompasses machine learning (ML), natural language processing (NLP), and other techniques developed using AI technology.
Automated aid	“A machine agent that performs a task that was previously and can still be completed by a human” (Kneeland et al., 2021, p. 335)
Chatbot, conversational agent, digital assistant, intelligent personal assistant, smart assistant, social robot, virtual assistant	“A computer program designed to simulate conversation with human users, especially over the Internet” (Adamopoulou and Moussiades, 2020, p. 373).
Computerized advice	Advice coming from a computerized decision-support system (Gillaizeau et al., 2013); see Decision aid/Decision support system
Decision aid/decision support system	“A combination of the information system and decision-making technology” (Yun et al., 2021, p. 285)
Robo-Advisor (in the context of financial investments)	“Automated online services that use computer algorithms to provide financial advice and manage customers’ investment portfolios” (Fisch et al., 2019, p. 13)

Existing terminology is relatively inconsistent, such that each row of this table cannot (and should not) be viewed as perfectly distinct from other rows. The various technologies listed here are covered to some extent (i.e., to the extent they are used in the AI advice literature) in the current review, under the rubric of AI advice. Keyword discrepancies between Tables 1 and 2 indicate that not every keyword search resulted in articles retained for inclusion.

In theory, the appeal of AI in decision-making is clear: an AI advisor has the potential to function as a “solution” to the cognitive and computational limits of the human mind, and hence to effectively and efficiently guide strategic organizational decision-making, which is an inherently complex and uncertain endeavor (Phillips-Wren, 2012; Burton et al., 2020; Trunk et al., 2020). Unfortunately, however, there exists a disconnect between advancements in AI-assisted decision-making and corresponding organizational research (Phan et al., 2017). In general, organizational research (e.g., research in industrial and organizational psychology and the closely related field of organizational behavior) has paid insufficient heed to the rapidly evolving field of algorithms and artificial intelligence (Phan et al., 2017; Kellogg et al., 2020), despite the increasing salience of such technologies in many organizational processes, from assisting with customer service and financial processes to diagnostic aids in flight management systems (Madhavan and Wiegmann, 2007; Lourenço et al., 2020; Vrontis et al., 2022). Although organizational research has begun to explore bureaucratic changes and structural responses to the introduction of AI (e.g., the implementation of AI-enabled employee recruiting practices; Hunkenschroer and Luetge, 2022), implications of the socio-cognitive influence of AI on employees and organizational systems have seldom been discussed. For instance, the developers of ChatGPT—an AI-driven natural language processing tool—are explicitly concerned about the risk that even the newest AI models will provide “harmful advice” (OpenAI, 2023). There also exist inconsistencies in organizational scholars’ understanding of how AI alters individuals’ gathering and usage of evidence for decision-making. For example, the introduction of AI may require new standards of evaluation for the processes and data used to make organizational decisions (Kellogg et al., 2020; Landers and Behrend, 2023).

This paper contributes to research and practice on AI advice to human decision makers in three main ways. First, the current research provides a conceptual framework through which to study advice from AI—thereby helping to summarize existing research, identify incongruous findings, and identify important areas in which existing research is sparse. Second, the current research draws on specific findings from the judgment and decision-making (JDM) literature to foster nuanced insights that can be beneficial to audiences in both psychology and AI research, rather than pitting them against each other. Third, the current research informs the development of AI that is compatible to a greater degree with human decision-makers than existing AI models [e.g., by facilitating human-AI “fit”; cf. Edwards (2008)], guides practitioners’ technical and design choices for AI advisors (Wilder et al., 2020; Lai et al., 2021; Inkpen et al., 2022), and more generally aids organizational policy and practice guidelines concerning advice from AI (e.g., by providing recommendations concerning how and when AI advice should be implemented in organizations). It also sheds light on what decision-makers need from AI advisors rather than focusing solely on the technological advancement of AI advisors (Lai et al., 2021), thereby mitigating the unintended detrimental aspects and effects of AI advice.

Thus, the broad purpose of the current work is to expand research on AI advice by examining existing research, and on that basis advancing a number of theoretical propositions, regarding how interactions of human decision-makers with AI advisors differ from or stay consistent with their interactions with human advisors.

**Figure 1:**
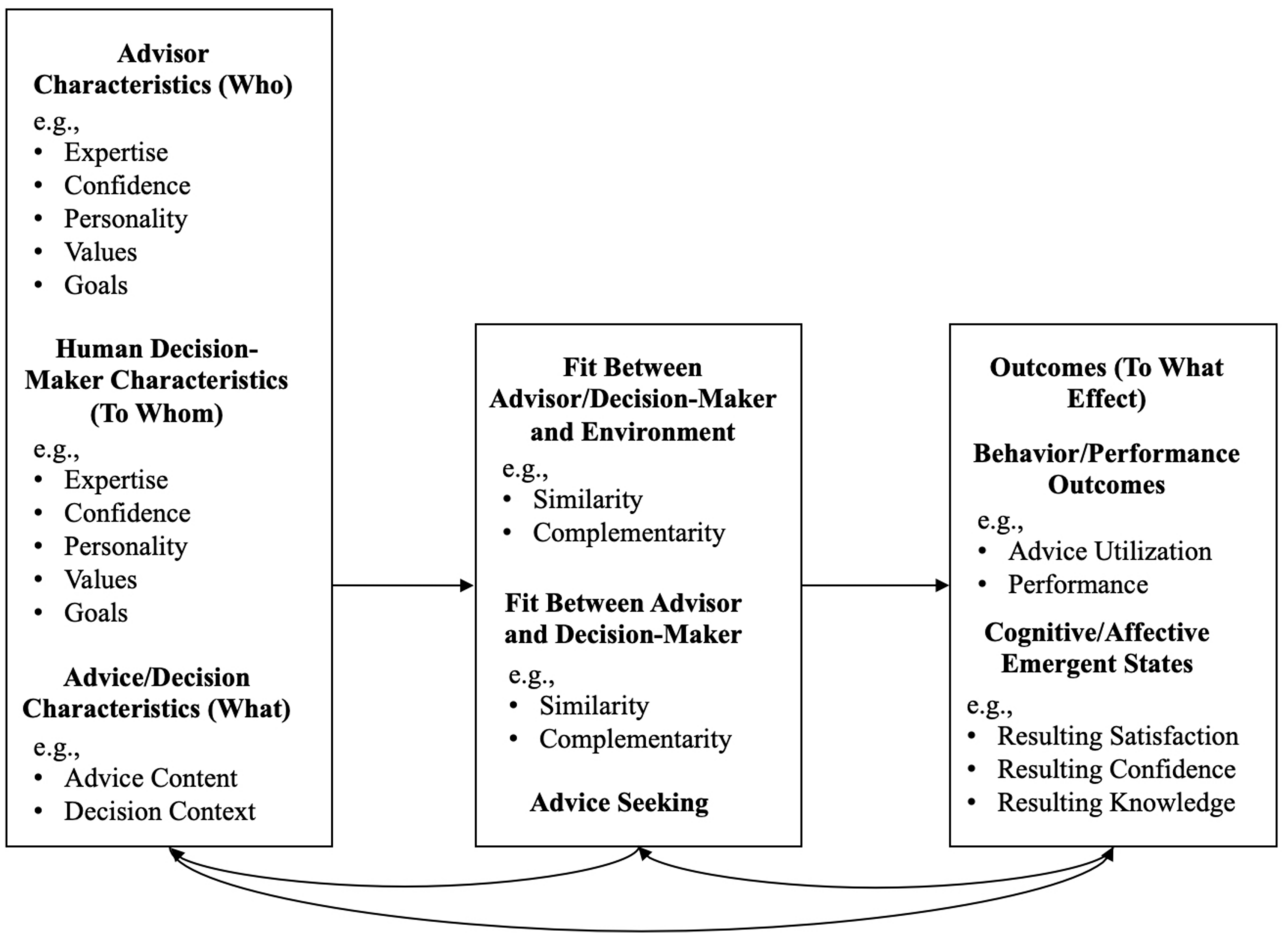
Conceptual AI Advisor – Human Decision-Maker – Situation Model. Advisor refers to the source of Al advice. For parsimony of terminology, and in the service of using the same terminology as that used in the organizational psychology/behavior literature on fit, here we consider the Al advisor, whether anthropomorphized or not, as a “person”.

We begin by defining key terms and explaining the scope of this review. We then present our conceptual model (see Figure 1), which adopts an AI-person-environment (here: AI advisor - human decision-maker - situation) fit framework modeled after person-environment and person-person fit frameworks in the organizational psychology/behavior literature (e.g., Edwards, 2008). This model organizes our research findings.

Each section of our research findings contains a summary of primary findings from the research we reviewed on a particular topic. To develop these section summaries, we drew on topics from the research literature on human advisors providing advice to human decision-makers. That research has mostly been conducted in the JDM field under the rubric of a “judge-advisor system.” Specifically, by first examining reviews of the human advice literature [see, in particular, Bonaccio and Dalal (2006) and Kämmer et al. (2023)], we extracted antecedents of advice (i.e., the determinants of advice solicitation) and outcomes of advice (i.e., the behavioral and performance outcomes of advice) as focal topics. For both antecedents and outcomes of advice, the literature on human advice discusses advisor characteristics, decision-maker characteristics, and environmental characteristics. Therefore, we followed suit by including subsections on each of these topics–and, within those subsections, focusing primarily on the specific characteristics identified in these literatures: for example, advisor confidence and expertise (Bonaccio and Dalal, 2006; Kämmer et al., 2023).

However, these topics obviously do not exist in isolation from each other. In particular, for the current review paper, the characteristics of the AI advisor interact with those of the human decision-maker, and the characteristics of both the AI advisor and the human decision-maker interact with the characteristics of the decision environment. To assess these interdependencies, we adopt frameworks from the organizational psychology research on person–person fit (to reflect the fit between the actual and the artificial “person,” in other words the human decision-maker and the AI advisor) as well as person-environment fit (to reflect the fit between the human decision-maker and the decision environment as well as the fit between the AI advisor and the decision environment). Finally, we elaborate on theoretical and practical applications of this research and explore future integrative research directions.

## 2 Conceptual boundaries

The definition of advice varies substantially across domains in terms of its content, specificity, and directiveness (MacGeorge and Van Swol, 2018). This may be explained to some extent by the potential consequences of advice in “almost every imaginable social and cultural context” (MacGeorge and Van Swol, 2018, p. 4). It may also be due in part to the relevance of advice as a construct across many academic disciplines such as psychology, communication, organizational behavior and human resource management, sociology, education, medicine, and public health (MacGeorge et al., 2016; MacGeorge and Van Swol, 2018). Despite this, the underlying theoretical “structure” of advice remains relatively consistent. Therefore, in this paper we use the following definition of advice [adapted from MacGeorge and Van Swol (2018)]: *advice is future-focused communication that focuses on the decision maker’s action, contains actual or apparent intent to guide the decision maker’s action* (i.e.*, behavior*)*, appears in the context of a decision or problem that makes action relevant, and may or may not involve some disparity in knowledge or expertise between advisor and decision-maker*.

In this paper, we focus specifically on advising interactions in which the human decision-maker receives advice from the AI advisor. As we discuss subsequently, there may be an imbalance in favor of the AI in terms of logical and computational abilities but a simultaneous imbalance in favor of the human in terms of social/communication abilities as well as ultimate responsibility for the decision. It should also be noted that, whereas AI has certainly advanced sufficiently to be able to accomplish actions independently, with minimal or no human input (Lai et al., 2021), these so-called performative AI or algorithms are not the focus of this review. There is also an intermediate case where the AI has a human overseer but acts independently unless and until it is overridden by the human. Those AIs are also not the focus of this review. Instead, this review focuses only on advisory AI, which provides input (advice) to the human decision-maker but does not act, instead leaving the decision to the human.

## 3 Method

**Figure 2:**
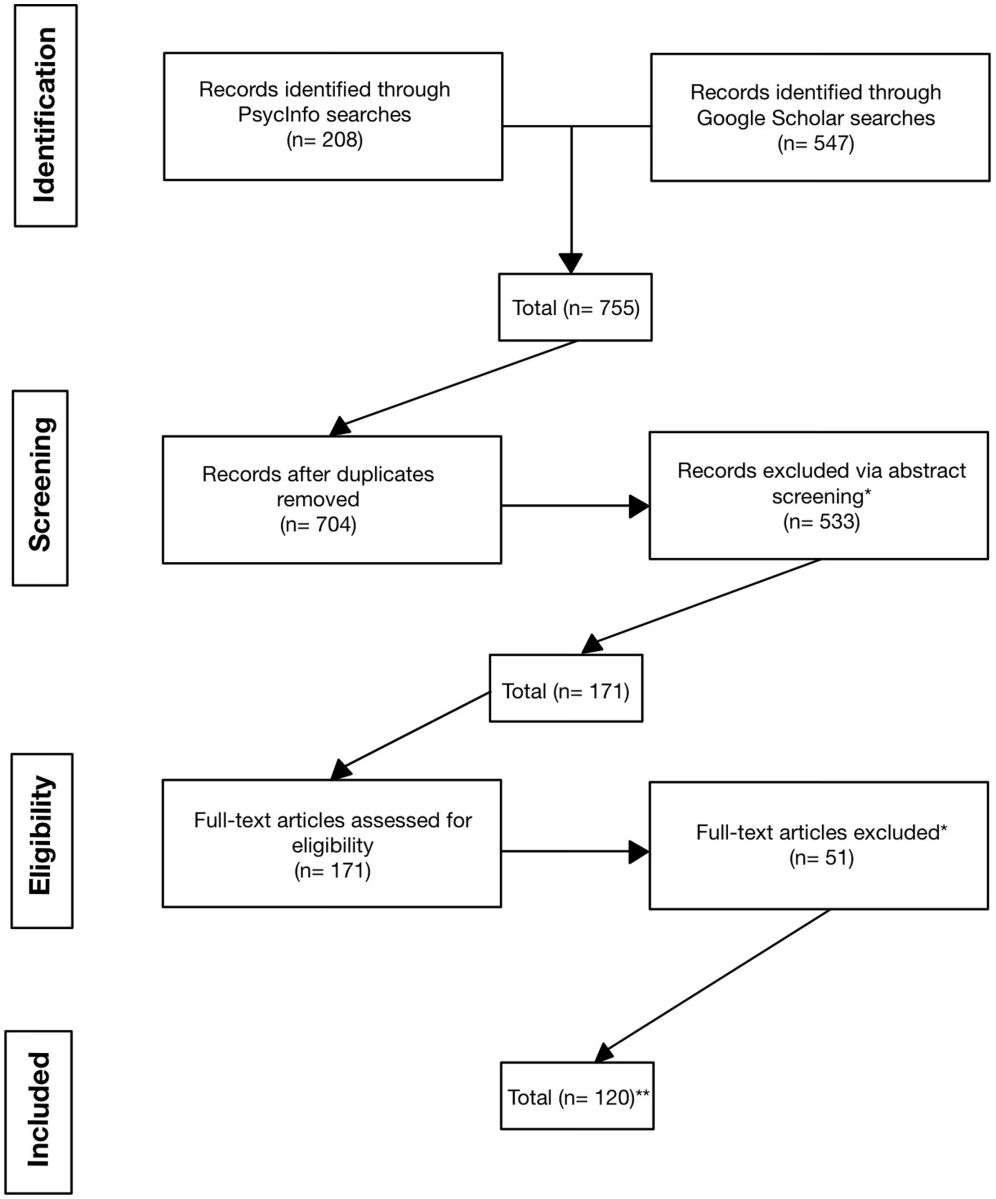
Preferred Reporting Items for Systematic Reviews and Meta-Analyses (PRISMA) Flow Diagram. *Articles excluded due to: advice is not a focal component of the study; study does not contain a focal human component; study is in a foreign language; full-text is not available. **The n of 120 reflects the initial number of studies reviewed. We also consulted a small number of additional sources in related areas throughout the development of the manuscript.

A review of literature on advice from AI was conducted using the online research platforms Google Scholar (principal resource) and PsycInfo (supplementary resource). Google Scholar and PsycInfo were searched using Boolean search terms comprising keywords that represented the intended content of the review. A list of search terms can be found in Table 2. Each keyword search was conducted using one keyword from the “Base” keywords in Table 2, the “and” operator, and one keyword from the “Technology” keywords in Table 2. In total, 755 articles were identified through primary searches, which were then screened for duplicates and for relevance to the study. Specifically, as regards relevance, articles were excluded if advice was not a focal component of the study, if the study did not involve human decision-makers and AI advisors, if the study was published in a language other than English, or if the full-text version of the article was not available. After screening, 120 articles were retained for primary coding. In the primary coding stage, authors coded articles for content in each of the categories from the conceptual model: AI advisor characteristics, human decision-maker characteristics, advice/decision characteristics, person-environment fit (i.e., fit between the decision-maker and the decision environment and between the advisor and the decision environment), person–person fit (i.e., fit between the advisor and the decision-maker), and outcomes of advice exchanges. See Figure 2 for a flow diagram of our inclusion and exclusion process.

**Table 2:** Literature search terms.

Keyword Domain	Keyword
Base	AdviceAdviseAdvisee Adviser Advising Advisor
Technology	AI Algorithm Artificial intelligence Automated aid Chatbot Computerized advice Conversational agent Cyber aid Decision aid Decision support system Digital assistant Intelligent personal assistant Robo-adviser Robo-advisor Smart assistant Virtual assistant Anthropomorphic agent*

We conducted systematic searches with each keyword from the “Base” keyword domain combined with each keyword from the “Technology” keyword domain. These searches were conducted across article titles in Google Scholar, and across article titles and abstracts in PsycInfo.*This search term was added as a follow-up after the initial searches had been conducted, and only across article titles. It did not result in any unique sources.

It should be noted that our goal in this paper was to review the literature on advice from AI and, in so doing, to draw a direct comparison between the human advice literature (primarily from the JDM field) and the AI advice literature, with the decision-maker being human in both cases. Thus, our literature search was specifically intended to address these questions. It is certainly true, however, that AI, and even human-AI interaction, is a very broad field. The present paper was not focused on instances where AI itself makes decisions without human intervention (i.e., performative algorithms; Jussupow et al., 2020), instances where human advice facilitates a decision made by an AI decision-maker (Enarsson et al., 2022), instances in which a work team is composed of some combination of human and AI members who must work together (Trunk et al., 2020; Sowa et al., 2021), and so forth. We also did not focus on topics such as algorithm (i.e., AI) aversion or appreciation (see Burton et al., 2020; Jussupow et al., 2020; Kaufmann et al., 2023) except insofar as they related specifically to advice from AI advisors. Therefore, we did not aim to comprehensively review the literatures in these other areas. Nonetheless, to inform the present review, we did consult the aforementioned sources in the current paragraph as well as a small number of additional sources (e.g., Jarrahi, 2018; Araujo et al., 2020; Zhang et al., 2023). Although there naturally exists some overlap in topics covered among the sources cited in this paragraph and the current review, the current review additionally covers several unique topics such as fit and framing effects as well as discusses human decision-maker reactions to AI in a way that does not limit itself to, let alone dichotomize into, aversion versus appreciation (see Figure 1). In sum, we believe our literature review strategy fit the goals of the review.

## 4 Research findings

This section reviews research findings associated with, first, the antecedents to advice and, second, the antecedents to the outcomes of advice (separately for behavior/performance outcomes and cognitive-affective outcomes). Within each of these domains, we discuss findings separately for advisor characteristics, decision-maker characteristics, and, where appropriate, environmental characteristics. Where possible, we begin by discussing research conclusions from the JDM literature involving human advisors—and we then discuss the extent to which these conclusions generalize to the case of AI advisors. Subsequently, we discuss the (thus far) small amount of research that has examined the important topic of the fit between the AI advisor, the human decision-maker, and/or the environment. The conceptual model, which organizes this section on research findings and additionally includes examples of the factors we discuss in the various portions of this section, is provided in Figure 1. A list of what we view as the most notable research findings is provided in Table 3.

**Table 3:** Summary of research findings by domain and subdomain.

Domain	Subdomain	Finding	Exemplary Citations
Antecedents of advice	Advisor characteristics	∙Certain characteristics may be judged or evaluated differently across human and AI advisors (e.g., competence may be judged differently based on decision context); however, these characteristics generally tend to influence advice solicitation in the same direction.	∙Gazit (2022), Hertz (2018), Hou and Jung (2021) and Longoni and Cian (2020)
	Decision-maker characteristics	∙Overconfident human decision-makers solicit less advice than those who are not overconfident from both AI and human advisors; however, it has been shown that decision-maker overconfidence can be somewhat ameliorated in the presence of an AI advisor (Logg et al., 2019). ∙Decision-maker anxiety *per se* increases advice-seeking from human advisors, whereas decision-maker technology anxiety decreases the perceived usefulness and acceptability of AI advisors.	∙Kämmer et al. (2023), Lewis (2018), Logg et al. (2019), and Willford (2021)∙Gino et al. (2012), Lindblom et al. (2012), Meuter et al. (2003)
Outcomes of advice: behavior/performance outcomes	Advisor characteristics	∙Perceptions of advisor expertise and perceptions of similarity (e.g., behavioral or demographic similarity) between advisor and decision-maker increase advice utilization from both AI and human advisors. In the case of AI advisors, “demographic” similarity comes into play when AI advisors are anthropomorphized. ∙Mistakes in past performance tend to be weighted more heavily against AI advisors than against human advisors.	∙Bonaccio and Dalal (2006), Lourenço et al. (2020), Schreuter et al. (2021), and Yaniv et al. (2011)∙Dietvorst et al. (2015), Prahl and Van Swol (2017)
	Decision-maker characteristics	∙Trust in the advisor increases advice utilization from both human and AI advisors; however, this trust may develop differently (i.e., based on different advisor characteristics) depending on whether the advisor is human or AI.	∙Bonaccio and Dalal (2006), Jung et al. (2018), and Sniezek and Van Swol (2001)
	Environmental characteristics	∙Decision-makers may be more receptive to human experts’ recommendations than AI advisors’ recommendations in situations that prompt an emotional response. ∙Framing of the advisor (e.g., introducing the advisor in a way that influences perceptions of competence) may explain divergent findings regarding the utilization versus discounting of AI advice.	∙Larkin et al. (2022), Longoni and Cian (2020)∙Castelo et al. (2019), Hou and Jung (2021)
Outcomes of advice: cognitive-affective outcomes	Advisor characteristics	∙Transparency and clarity of design influence decision-makers’ satisfaction with AI advisors; however, the impact of transparency on satisfaction may be mediated by the perceived value of advice.	∙Jung et al. (2018) and Willford (2021)
	Decision-maker characteristics	∙Higher decision-maker numeracy (i.e., ability to understand probability and other numerical concepts) is associated with better reactions to AI advice.	∙Logg et al. (2019) and Willford (2021)
	Environmental characteristics	∙When environmental characteristics are seen as fitting for the advisor (e.g., lay beliefs regarding the suitability of AI for objective vs. subjective tasks), better cognitive and affective outcomes such as trust and satisfaction are likely to accrue to the human decision-maker.	∙Hertz (2018) and Longoni and Cian (2020)
Fit between the advisor, decision-maker, and environment		∙Perceived human-like traits and/or abilities (e.g., the ability to make moral judgments) in the AI advisor (e.g., when anthropomorphized) can increase decision-maker trust in and advice utilization from the AI advisor. ∙Complementarity in expertise between the advisor and decision-maker (with advisor expertise being higher) fosters advice utilization.	∙Hertz (2018), Pak et al. (2012)∙Inkpen et al. (2022), Zhang et al. (2022)

The individual sections of the paper in which these findings are discussed provide more detailed (and contextualized) explanations, including in some cases our speculation regarding the durability (or lack thereof) of the findings in light of rapid advances in AI.

### 4.1 Antecedents of advice

The first step of an advising interaction includes the antecedents of advice. Research on the antecedents of advice primarily examines the individual determinants of advice solicitation. We note here that, in contrast to advice *solicitation*, advice *utilization* is an outcome (specifically, a behavior/performance outcome) of advice. Thus, the antecedents to advice solicitation are discussed in this section; in contrast, the antecedents to advice utilization are discussed in a later section.

#### 4.1.1 Advisor characteristics

Reviews of the human advice literature maintain that several advisor characteristics play an important role in the extent to which decision-makers solicit advice from them (Bonaccio and Dalal, 2006; Lim et al., 2020; Kämmer et al., 2023). We discuss the role of these advisor characteristics when the advisor is not human but AI.

##### 4.1.1.1 Competence

Perceived competence on the part of the human advisor (e.g., advisor expertise, experience, training, or credibility) increases advice solicitation by the decision-maker (Porath et al., 2015; Lim et al., 2020; Kämmer et al., 2023), as does perceived competence on the part of the AI advisor (Hou and Jung, 2021; Gazit, 2022). However, competence may be judged differently based on the decision context. This is due to anticipated differences in skill requirements for social contexts versus analytical ones: human advisors may be considered more competent in judging emotions, whereas AI advisors may be considered more competent in technical or mathematical tasks (Hertz, 2018; Longoni and Cian, 2020). For instance, Hertz (2018) showed that human advisors were preferred (i.e., selected as a source for advice) for a task in which participants were asked to identify the emotion being experienced by a human in a photograph, whereas AI advisors were preferred for a task in which participants were asked to complete an addition or subtraction operation.

Although the existing research suggests that AI advisors are typically not seen as competent in judging emotions, we note that significant advances in technology have allowed some recent AI systems to effectively capture subtle expressions of emotion and other physiological signals. These systems use advanced technologies and machine learning to analyze patterns in facial expressions, voice intonations, word usage, sentence structure, and body movements to determine the emotional state of a person (Turabzadeh et al., 2018; Nandwani and Verma, 2021; Joshi and Kanoongo, 2022). However, accuracy (and usability) in emotion detection likely still require significant improvement if the goal is for AI advisors to be perceived as highly emotionally competent in an affective decision-context. For example, intricacies such as grammar and spelling errors, the use of slang, and lack of clarity and context in human writing and speech can limit the ability of machines to perform sentiment and emotion analysis (Nandwani and Verma, 2021).

##### 4.1.1.2 Trustworthiness

Trust has often been studied in research on human-human advice exchanges (Bonaccio and Dalal, 2006; Kämmer et al., 2023), and is typically seen as stemming from the perceived ability, benevolence, and integrity of the “trustee” [i.e., entity being trusted; Mayer et al. (1995)]. Trust in human advisors increases advice-seeking [and advice utilization, as discussed in later sections; Sniezek and Van Swol (2001) and Dalal and Bonaccio (2010)], as does trust in AI advisors. Importantly, however, the trustworthiness of AI advisors is not completely parallel to that of human advisors. There are differences in attribution processes and differences in the assessment of predictability and dependability (Rempel et al., 1985; Madhavan and Wiegmann, 2007). Further, it has been argued that AI cannot satisfy the conditions of normative principles such as moral agency and moral responsibility – factors that may be used in the evaluation of advisor trustworthiness. Thus, caution must be exercised when considering these criteria in the context of AI advisors versus human advisors (Hakli and Mäkelä, 2019).

Perceived trustworthiness of an AI advisor may arise from factors such as system characteristics (e.g., the reliability of the system) and perceived credibility (e.g., perceived expertise; Madhavan and Wiegmann, 2007)—factors also applicable to human advisors. Yet, perceived trustworthiness of an AI advisor may stem from aspects of the AI such as transparency and explainability, technical robustness, privacy and data governance, and so forth (Walsh et al., 2019; Linardatos et al., 2020)—factors inapplicable to human advisors. For example, participants may use the degree of AI usability or interpretability as a cue for AI trustworthiness (Jung et al., 2018; Linardatos et al., 2020). An aspect of trust in AI advisors that is difficult to compare to traditional models of trust is the influence of anthropomorphization. Research has shown that trust increases as anthropomorphism increases (Pak et al., 2012), and that AI advisors are perceived as more trustworthy when they have a human-like appearance as compared to a mechanical appearance (Madhavan and Wiegmann, 2007). This is perhaps due to the subconscious application of human-human social interaction rules or norms that lead to the perception of AI as more trustworthy (Madhavan and Wiegmann, 2007).

##### 4.1.1.3 Personality

Relatively few studies have examined the influence of human advisor personality on advice-seeking, despite existing studies having found promising results [Bonaccio and Dalal, 2006; see also Lim et al. (2020)]. This scarcity appears to an even greater extent in the AI advice literature. Some exceptions include research on AI advice that occurs via chatbot, which indicates that decision-makers prefer seeking advice from AI advisors that convey humor and positive “personality” (Lucien, 2021; Kuhail et al., 2022). Völkel and Kaya (2021) found specifically that chatbots exhibiting high agreeableness were more likely to attract human users. It should be noted that the issue of AI personality is likely to increase in importance in the immediate future (e.g., Bing’s ChatGPT-enabled search already allows human users to set a specific “personality” for the AI search), consequently making this an important area for future research to investigate in the context of AI advisors.

##### 4.1.1.4 Appearance of advisor

The appearance of the advisor can also influence advice-seeking behaviors. Although this topic has not been discussed much in research on advice from humans, it is an important topic in research on advice from AI, and in research comparing advice from AI and human advisors. Specifically, Hertz (2018) found that the human-likeness of the agent significantly influenced advice seeking between human and AI advisors such that nonhuman agents were less likely to be chosen as advisors for social tasks than for analytical tasks. However, the effects of anthropomorphization or human-likeness may not be linear: if the appearance of the AI advisor is *too* human-like, the effects of the so-called “uncanny valley” may come into play: decision-makers may be turned off because the chatbot seems *very*, yet not *completely*, human-like (Duffy, 2003; Lucien, 2021; see also Gray and Wegner, 2012).

#### 4.1.2 Decision-maker characteristics

The characteristics of the decision-maker also play an important role in the extent to which they decide to solicit advice (Kämmer et al., 2023).

##### 4.1.2.1 Confidence

It is well-established by the human advice literature that human decision-makers tend to be disproportionately confident in their own judgments as compared to their advisors’ judgments, a phenomenon sometimes referred to as “egocentric advice discounting” (Bonaccio and Dalal, 2006). Specifically, when asked to choose between their own judgment and that of a peer, decision-makers will disproportionately choose their own judgment. This schema reduces solicitation of advice; it has been shown that decision-makers who are overconfident (having more confidence than warranted in their own abilities; Sniezek and Buckley, 1995) solicit advice to a lesser extent than those who are not overconfident from both human (Kämmer et al., 2023) and AI (Lewis, 2018; Willford, 2021) advisors. Interestingly, using an estimation task in which decision-makers were asked to rank U.S. states in terms of number of airline passengers, Logg et al. (2019) found that when the “peer” is an algorithm, decision-makers appropriately judge the algorithm’s advice as better than their own opinion, demonstrating that the presence of an AI or algorithmic decision-maker can serve to ameliorate some facets of decision-makers’ overconfidence bias.

##### 4.1.2.2 Anxiety

On a related note, the human advice literature has found that decision-makers who are experiencing incidental anxiety are more likely to solicit advice (Gino et al., 2012). In the AI advice literature, in contrast, research has found that feeling anxious about using technology increases technological mistrust and decreases perceived usefulness and acceptability (Meuter et al., 2003; Lindblom et al., 2012). This anxiety could arise in part from individuals’ perceived (in) ability to successfully use AI. This constitutes an interesting divergence in the human and AI advice literature: whereas anxiety *per se*, or anxiety about the decision, has been found to increase advice solicitation from human advisors, anxiety about technology in particular may decrease advice solicitation from AI advisors. These findings support the need for more domain-specific measures of anxiety (e.g., anxiety about technology or, even more specifically, about AI) to clarify the influence of anxiety on advice-seeking from AI.

##### 4.1.2.3 Personality

Regarding other decision-maker characteristics, findings from the human advice literature on personality indicate that individuals who score high on conscientiousness and agreeableness, and low on neuroticism, tend to have higher advice-seeking tendencies (Battistoni and Colladon, 2014; Chatterjee and Fan, 2021). Furthermore, findings from the human advice literature in the domain of financial advice demonstrate that decision-maker extraversion is negatively associated with financial advice seeking, and decision-maker conscientiousness and openness are positively associated with financial advice seeking (Chatterjee and Fan, 2021). Conversely, a study on the impact of human personality on robo-advisor usage found that personality traits do not consistently affect the use of the robo-advisor (Oehler et al., 2022). More research is therefore needed to compare the extent to which decision-maker personality exerts similar versus different effects on advice-seeking from humans versus AI.

### 4.2 Outcomes of advice

Research on the outcomes of advice most commonly examines the individual and environmental determinants of behavioral and performance outcomes of advice, such as advice utilization by the decision-maker (Bonaccio and Dalal, 2006). The research reviewed in the following section therefore begins by discussing the advisor, decision-maker, and environmental characteristics that influence behavioral and performance outcomes of advice. We subsequently review the determinants of the less commonly studied cognitive-affective outcomes of advice, such as decision-maker and advisor satisfaction and confidence resulting from the advising interaction.

#### 4.2.1 Behavior/performance outcomes

Given the prevalence and significance of the decision-maker’s advice utilization as a behavioral outcome of advice (Bonaccio and Dalal, 2006; Kämmer et al., 2023), much of the following section discusses advice utilization, defined simply as the extent to which the decision-maker follows the advisor’s advice (Bonaccio and Dalal, 2006). However, we also review additional behavior/performance outcomes such as the decision-maker’s intention to seek advice again (i.e., on future decisions).

##### 4.2.1.1 Advisor characteristics

Advisor characteristics play an important role in determining behavioral and performance outcomes, such as the extent to which decision-makers utilize advice from others (Kämmer et al., 2023).

###### 4.2.1.1.1 Expertise

Perceptions of advisor expertise increase advice utilization by the decision-maker in the case of both human advisors (Bonaccio and Dalal, 2006) and AI ones (Lourenço et al., 2020; Hou and Jung, 2021; Mesbah et al., 2021). Although advisor expertise is defined here as the knowledge,^2^ skills, and abilities of the advisor in a particular domain, decision-makers may evaluate the expertise not just of the AI advisor but also of the developer and/or provider of the AI (i.e., a human or an organization consisting of humans; Lourenço et al., 2020; Bianchi and Briere, 2021). For example, in a study using a retirement investment task, Lourenço et al. (2020) found that advice utilization was influenced by the perceptions of trust and expertise that decision-makers formed about the firm providing the AI advice.

In terms of decision-maker preferences between human and AI advisors, some research has found that decision-makers prefer human over AI advice (e.g., Dietvorst et al., 2015; Larkin et al., 2022). This is one form of what is often referred to as algorithm aversion, or general negative attitudes and behaviors toward the algorithm (Logg et al., 2019; Lai et al., 2021). For example, Larkin et al. (2022) found that participants indicated they would prefer to receive recommendations from a human expert versus AI in financial and, even more so, healthcare and contexts. Similarly, Dietvorst et al. (2015) found that, across forecasting tasks on student performance and airline performance, decision-makers consistently chose human judgment when choosing between AI forecasts and either their own forecasts or the forecasts of another human participant.

However, other research has found the converse (e.g., Logg et al., 2019; Kennedy et al., 2022). For example, Kennedy et al. (2022) found that in geopolitical and criminal justice forecasting experiments, decision makers placed a higher weight on AI advice (i.e., forecasting algorithms) relative to several kinds of human advice (i.e., aggregate of expert decision-maker responses; aggregate of non-expert decision-maker responses)–in other words, algorithm appreciation rather than aversion.

Although the decision to use or not use AI advice is often labeled as algorithm aversion or algorithm appreciation, this can be an oversimplification. This preference is likely influenced by several relevant factors. For instance, the factors listed above (e.g., the context of the decision, the presence of another advisor) can influence the decision to use or not use advice. The Dunning-Kruger effect, referring to the overestimation of one’s own competence or expertise (Dunning, 2011), may also cause people to overestimate their own abilities. The fact that people tend to overweight their own opinion compared to external sources of information likely holds true across human and AI (i.e., algorithmic) sources of advice, which could explain some instances of so-called “aversion” in which a human decision-maker is asked to choose between their own forecast and the recommendation of an AI advisor (e.g., Dietvorst et al., 2015).

###### 4.2.1.1.2 Distance of recommendations

The distance between the advisor’s recommendation and the decision-maker’s own initial (pre-advice) judgment also impacts advice utilization. In human advice exchanges, the weight that decision-makers place on advice increases when advisor estimates are neither too close to nor too distant from the decision maker’s initial estimate (Moussaïd et al., 2013; Schultze et al., 2015; Ecken and Pibernik, 2016; Hütter and Ache, 2016). Using a laboratory estimation task, a study on AI advice showed that decision-makers are more likely to follow expert AI advisors if the advisors’ recommendations are close to the decision-makers’ own initial judgments (Mesbah et al., 2021). Overall, the AI advice literature should examine this issue with more granular conceptualizations of distance, so as to see if results are consistent with the human advice findings.

###### 4.2.1.1.3 Past performance

Another advisor characteristic that influences behavioral and performance outcomes (specifically, advice utilization) is the past performance—that is, decision accuracy—of the advisor (Fischer and Harvey, 1999; Bonaccio and Dalal, 2006). Indeed, decision-makers’ perceptions of advisor expertise often occur as a joint effect of the advisor’s past performance and status (Önkal et al., 2017). Despite the importance of past performance in judgments of human expertise and decisions to use advice from humans, some research has shown that evidence supporting the efficacy of AI advice (i.e., past AI advisor performance) does little to reduce resistance to utilizing their advice (Dietvorst et al., 2015). Research has also shown that decision-makers place more weight on AI errors than human errors (Dietvorst et al., 2015; Prahl and Van Swol, 2017; Gaube et al., 2023). For instance, Dietvorst et al. (2015) found that individuals were less likely to use AI advice after it made a mistake, despite its performance remaining higher than its human advisor counterpart. Further, Prahl and Van Swol (2017) found that the experience of “bad” advice (i.e., advice that decreases decision-maker accuracy) made decision-makers more reticent to use AI advisors. This phenomenon can also be seen in popular culture: after the Google AI chatbot Bard gave an incorrect answer when it was first unveiled to the public, its stock value plummeted (Guardian News and Media, 2023).

Several potential explanations for this phenomenon can be drawn from literature on human judgment and decision making. For example, the schema that AI should perform perfectly and without mistakes (the “perfection schema”; Madhavan and Wiegmann, 2007) suggests that trust in the AI advisor decreases rapidly due to the belief that AI should be perfect whereas humans are likely to make mistakes. This may lead to AI mistakes having a higher likelihood of being noticed and remembered than human mistakes, because AI mistakes are in opposition to the existing perfection schema (Madhavan and Wiegmann, 2007). An additional explanation is that human decision-making processes may be seen as adaptable, whereas AI decision-making processes may be seen as more immutable. This leads to the assumption that, whereas a human advisor has the ability to detect and correct mistakes, mistakes from an AI advisor may suggest a fundamental flaw in the system—and therefore small mistakes from an AI advisor are more likely to result in global negative judgment of the AI’s abilities, relative to mistakes from a human advisor (Dietvorst et al., 2015). Recent research supports this explanation: it was shown that demonstrating an AI advisor’s ability to learn reduces resistance to using its advice (Berger et al., 2020). These findings support the idea that AI and human advisors are subject to distinct recipient biases and response tendencies (Madhavan and Wiegmann, 2007). Accordingly, erroneous AI advice may more strongly undermine a decision-maker’s trust than erroneous human advice; AI mistakes tend to be weighted more heavily, even when the AI statistically outperforms a comparable human advisor.

###### 4.2.1.1.4 Transparency

An additional advisor characteristic that impacts advice utilization by decision-makers is the amount of access that advisors provide to their reasoning and decision process. Specifically, research on human advice has contended that advice discounting may occur partially due to decision-makers’ lack of access to their advisors’ internal justifications and evidence for formulating advice (Bonaccio and Dalal, 2006). Thus, a parallel may be drawn here: a lack of access to and understanding of the underlying computational processes of AI advisors may reduce decision-makers’ likelihood to utilize the AI advice (Linardatos et al., 2020).

Although much research suggests the benefits of transparency of AI advice in terms of the cognitive/affective outcomes of advice (as discussed in a subsequent section), transparency has also been studied with regard to advice utilization (the current focus), with mixed findings. Specifically, some research has found that transparency does not always increase decision-maker advice utilization (Willford, 2021; Lehmann et al., 2022). For instance, Lehmann et al. (2022) found that the impact of transparency on advice utilization is mediated by the extent to which participants perceive the advice to be valuable, such that participants who interact with a transparently designed algorithm may underestimate its utility (value) if it is simple but accurately estimate its utility if it is complex (Lehmann et al., 2022). Willford (2021) also found that participants who interacted with transparent AI relied on it less. This supports the idea that if transparency leads to a lower evaluation of the AI advisor’s utility (i.e., if, once the metaphorical “black box” is opened, what lies inside no longer seems impressive), it does not increase advice utilization. A different explanation proposed by You et al. (2022) suggests that the occasionally negative influence of transparency on advice utilization may stem from increased cognitive burden—that is, information provided about AI functioning is complex to the extent that it introduces a detrimentally high cognitive load. Future research should therefore study the circumstances under which AI transparency yields positive versus negative effects—and, in cases involving negative effects, which explanation receives more support.

##### 4.2.1.2 Decision-maker characteristics

Decision-maker characteristics also play an important role in determining behavioral and performance outcomes, such as the extent to which decision-makers utilize advice from others (Kämmer et al., 2023).

###### 4.2.1.2.1 Trust

Trust can occur both as a propensity to trust, which refers to the idea that some individuals are in general more likely to trust than others, and as a momentary evaluation, which refers to the idea that any individual may be more likely to trust in some situations than in others (Mayer et al., 1995). Overall, decision-maker trust increases utilization of advice from both human advisors (Sniezek and Van Swol, 2001; Bonaccio and Dalal, 2006) and AI advisors (Wise, 2000; Jung et al., 2018; Cho, 2019; Rossi and Utkus, 2020).

Decision-maker trust can, however, develop differently for humans and for AI, perhaps partially as a result of the different attribution processes that decision-makers engage in for human versus AI advisors (Madhavan and Wiegmann, 2007). For example, trust can be developed on the basis of perceived ability, benevolence, and integrity for human advisors, versus on the basis of the degree of AI usability or interpretability for AI advisors (Walsh et al., 2019; Linardatos et al., 2020).

Although further developments in AI may not change the positive influence of decision-maker trust in the advisor on advice utilization, decision-maker trust in AI advisors, *per se*, may be expected to increase over time. Additionally, unlike for human advisors, the aspects of AI advisors that influence decision-maker trust may be relatively easy to manipulate (Glikson and Woolley, 2020). Therefore, as advances in our understanding of the features of AI that influence trust continue to advance, designing AI to foster trust is likely to become increasingly common and effective.

##### 4.2.1.3 Environmental characteristics

The advice environment also impacts advice utilization by decision-makers: utilizing advice from an AI (or human) advisor is substantially influenced by context, for example task type and difficulty (Hertz, 2018), and decision significance (Saragih and Morrison, 2022). Further, situations that elicit affective versus utilitarian processing may impact the degree to which a decision-maker is likely to take advice from a human or AI advisor.

###### 4.2.1.3.1 Affective situational demands

Some research suggests that people may be more receptive to human experts’ recommendations than AI recommendations in situations that prompt an affective response [e.g., assessing how enjoyable a real estate investment would be or how pleasant something tastes; Longoni and Cian (2020) and Larkin et al. (2022)]. This idea is related to the “word of machine” effect, a lay belief that humans possess greater expertise in hedonic domains, whereas AI possesses greater expertise in utilitarian domains (Longoni and Cian, 2020). These ideas are corroborated by experimental research on the acceptance of AI advice in objective numerical tasks versus emotionally driven or subjective tasks (Castelo et al., 2019; Gazit, 2022): people judge the suitability of the environment to the perceived capabilities of the advisor (human vs. AI; Vodrahalli et al., 2022) and utilize or discount advice accordingly. However, this may not always be the case: Logg et al.’s (2019) findings that algorithmic advice is preferred even when predicting interpersonal attraction (a presumably emotion-driven task) suggest that broad categorizations of task type may be insufficient to predict discounting versus utilization.

###### 4.2.1.3.2 Framing

Recent research suggests that how the AI is introduced (i.e., “framing”) may explain divergent findings on the choice to utilize or discount AI advice (Hou and Jung, 2021). In particular, the framing of the advisor can influence its perceived competence, which then influences the attractiveness of the advice it is proffering. Framing can be achieved through various means aimed at influencing judgments of competence: for example, providing prior performance data for both human and AI advisors, listing domains of high versus low competence for both human and AI advisors, providing the educational/training qualifications of human advisors, listing the types of human users (themselves with high or low competence) of AI advice, and so forth (Hou and Jung, 2021). Thus, the effect of task type is likely strongest when the perceived competence differential (due to framing) between the human and AI advisor is small.

The stability of these findings as AI continues to advance may depend in part on the speed with which technology develops its ability to communicate and respond in a human-like manner across both affective and utilitarian contexts. The popularity and advancements of GPT-3, GPT-4, and other AI language models suggest that these developments are occurring at an extremely rapid pace (Floridi and Chiriatti, 2020) as scientists continue to acquire insights that support the improvement of future model versions (Binz and Schulz, 2023). Specifically, new advancements in AI demonstrate that models are developing the ability to solve complex reasoning problems in addition to generating language and predictions (Binz and Schulz, 2023). Importantly, AI systems have begun to be capable of determining an individual’s emotional state via analysis of facial expressions, voice intonations, word usage, sentence structure, and body movements (Turabzadeh et al., 2018; Nandwani and Verma, 2021; Joshi and Kanoongo, 2022). Therefore, the decision-maker’s perception of discrepancies between the abilities of human versus AI advisors—particularly in affective and/or emotionally driven tasks—is likely to decrease over time.

#### 4.2.2 Cognitive-affective outcomes

In this section, we discuss the factors affecting cognitive-affective outcomes of advice, beginning with the impact of advisor characteristics and then moving on to decision-maker and environmental characteristics. It is noteworthy that the cognitive and affective outcomes of advice exchanges (e.g., advisor and decision-maker satisfaction, increased knowledge, and increased confidence) are far less commonly researched and discussed than the behavioral and performance outcomes of advice exchanges discussed previously (e.g., advice utilization and decision accuracy). However, the implications of cognitive and affective outcomes of advice are significant, perhaps particularly in the context of human reactions to AI advice, and therefore it is important to study the factors that influence these cognitive and affective outcomes.

##### 4.2.2.1 Advisor transparency

In terms of advisor characteristics that influence the cognitive and affective outcomes of advice, transparency and clarity of design have been demonstrated to influence decision-makers’ satisfaction with AI advisors (in addition to decision-makers’ advice utilization, which was covered previously, under the behavior/performance outcomes of advice). In fact, a significant amount of attention has been given to the “black box” nature of AI and algorithms (Rudin, 2019; Burton et al., 2020; Linardatos et al., 2020): it has been claimed that black-box AI/algorithms lead to algorithm aversion whereas information transparency and better user interface leads to higher satisfaction with AI/algorithmic advisors (Jung et al., 2018). The increasing complexity of AI (Linardatos et al., 2020) suggests that fostering transparency and clarity needs to be a primary focus of AI developers as they seek to improve the performance of their models and systems. This relationship is nuanced, however: a complex AI accompanied by a simple explanation may result in decision-maker skepticism, as individuals generally expect complex systems to have complex explanations (Bertrand et al., 2022). Therefore, despite the intelligibility of simpler explanations, it has been recommended that AI advisor developers should focus on providing coherent and broad explanations, with a focus on scope over simplicity (Bertrand et al., 2022). Generally, this area of research suggests that developers of AI should seek to find the balance between performance and interpretability that best serves individuals and organizations, thereby providing AI that is trustworthy, fair, robust, and high performing (Linardatos et al., 2020). For example, an AI that is intended to aid organizational Human Resources personnel in the scoring of virtual asynchronous interviews by job applicants should have clarity surrounding the input data (job incumbent data), model design (relevance of included predictor variables), model development (documentation of model creation), model features (the natural language processing approaches adopted), model processes (the model tests that were conducted), and model outputs (whether scores are reliable and valid; Landers and Behrend, 2023).

##### 4.2.2.2 Decision-maker individual differences

Research on the influence of decision-maker characteristics in human advice has been limited, with some research demonstrating that individual differences in preferences for autonomy influence reactions to advice (Koestner et al., 1999; Bonaccio and Dalal, 2006). For AI advice, on the other hand, older decision-makers are generally less satisfied with AI advice than younger decision-makers (Lourenço et al., 2020)—a trend likely due to differences in familiarity with technology rather than age *per se*. Further, these authors found that women on average were less satisfied than men with the AI advice they received. This is also potentially related to differences in familiarity with technology; these authors found that women tended to perceive themselves as having less user expertise than men. Research has also found that higher decision-maker numeracy (i.e., one’s ability to understand probability and numerical concepts; Peters et al., 2006) tends to correlate with a better reaction to AI advice (Logg et al., 2019; Willford, 2021). Interestingly, despite findings regarding the impact of numeracy on reactions to AI advice (Logg et al., 2019; Willford, 2021), research on education level has revealed mixed findings. For instance, a study on financial robo-advice found that more highly educated individuals were less trusting and somewhat less satisfied with the advice than less highly educated individuals (Lourenço et al., 2020). Conversely, however, a study on individuals’ trust of public policy AI (e.g., AI used for predicting criminal recidivism and political events; Kennedy et al., 2022) found that individuals with more education gave more weight to AI advice. Yet another study (Saragih and Morrison, 2022) found that there were no significant differences between AI adoption rate for those who were highly educated versus those who were not.

Future research should therefore examine a wide variety of factors simultaneously in an attempt to distinguish the underlying causes from the confounding variables with which the underlying causes are correlated. For example, as alluded to previously, decision-maker age is most likely correlated negatively with decision-maker familiarity with technology, with the latter rather than the former potentially being the underlying driver of satisfaction with AI advice. Additionally, the intercorrelations among factors may matter more in some contexts than others. For example, decision-maker education level is most likely correlated positively with decision-maker income/wealth, with the underlying driver of satisfaction with AI advice perhaps being the latter in financial decisions but the former in decisions involving which books to read.

##### 4.2.2.3 Environmental characteristics

Environmental characteristics are also likely to influence decision-makers’ cognitive and affective reactions to advice. Whereas, as noted above, aversion to versus appreciation of AI advice often functions as an *antecedent* to focal behavioral and/or performance outcomes of advice interactions (e.g., advice utilization), it may also arise as a cognitive-affective *outcome* of an advice interaction between a human decision-maker and an AI advisor (e.g., as a result of seeing the advisor err; Dietvorst et al., 2015). If the environmental characteristics (in this case, task characteristics) are seen as fitting for the advisor, there are likely to be better cognitive and affective outcomes on the part of the human decision-maker, such as trust and satisfaction. Developments in AI portend well for as-yet understudied research domains, such as the influence of environmental characteristics on cognitive-affective reactions to advice. Given that decision-makers’ reactions to AI advice are likely a result of many complex interactions between themselves, their AI advisors, and the decision environments, research that uncovers the specific reasons for discrepant findings regarding decision-maker reactions to AI advice will allow organizations to more productively involve AI in their decision-making processes.

### 4.3 Fit between the advisor, decision-maker, and situation

To aid our examination of characteristics that similarly or differentially impact human and AI advice exchanges and outcomes, we draw on person–person (i.e., interpersonal) and person-environment fit theory (Edwards, 2008). Fit refers to the compatibility that occurs when characteristics are well-matched between a person and either another person or the environment (Kristof-Brown et al., 2005). Whereas supplementary fit refers to similarity between an individual and another individual or else the environment, such that similarity is assumed to have positive effects, complementary fit refers to a difference between an individual and another individual or else the environment, such that the weakness of one is *complemented* by the strength of the other (Edwards, 2008). In the context of AI advice exchanges, “fit” may describe “person”-person fit (i.e., the fit between the AI advisor and human decision-maker), or “person”-environment fit (i.e., AI advisor-environment fit or human decision-maker-environment fit).

#### 4.3.1 Similarity

Similarity on some characteristics between advisor and decision-maker is consequential in JDM contexts. For example, perceived human-like traits and/or abilities (e.g., the ability to make moral judgments) in the AI advisor can increase decision-maker trust in and advice utilization from the AI advisor (Madhavan and Wiegmann, 2007; Pak et al., 2012; Hertz, 2018). Some studies have also shown that trust can be fostered via similarity of other demographic characteristics such as age, gender, ethnicity, and voice between humans and anthropomorphized AI [Muralidharan et al., 2014; Verberne et al., 2015; De Visser et al., 2016; for analogous results regarding similarity in the human advisor literature, see Lim et al. (2020)]. Specifically, Muralidharan et al. (2014) showed that human-like speech had higher trust ratings than machine-like speech, and Verberne et al. (2015) demonstrated that perceptions of artificial agents’ trustworthiness increased with displays of facial similarity, mimicry, and shared goals. An additional positive implication of similarity in human-likeness is that it may decrease the trust breakdown (e.g., after a mistake by the advisor) that occurs more strongly for AI advisors than for human advisors (De Visser et al., 2016).

#### 4.3.2 Complementarity

For other characteristics, complementarity is of greater value than similarity. For instance, complementarity in expertise between the advisor and decision-maker (with advisor expertise being higher) fosters advice utilization (Zhang et al., 2022; Gaube et al., 2023). More specifically, Zhang et al. (2022) found that human decision-makers detect and utilize AI advice more when it is complementary to their own expertise; however, they did not always trust the AI advisor more. The authors suggest that the developers of AI advisory systems should prioritize the ability to assess and cater to the expertise of the human decision-maker, such that complementarity can be reached. Gaube et al. (2023); see also Dell'Acqua et al. (2023) and Noy and Zhang (2023) found that non-task experts may be especially likely to benefit from AI advisors (in their case, medical decision-support systems).

In further support of this idea, recent findings on human-AI collaboration showed that a user’s baseline expertise impacts the effectiveness of collaboration between humans and AI, and that tuning (i.e., adjusting AI properties) can positively impact human-AI performance after taking user (i.e., human decision-maker) characteristics into account (Inkpen et al., 2022) and/or by taking environmental characteristics of decisions into account. Specifically, Inkpen et al. (2022) suggest that tuning the true positive and true negative rates of AI recommendations can help optimize human-AI complementarity. This is most beneficial when the tuning is aligned with decision-makers’ strengths and weaknesses. For example, decision-makers who were mid-performing were best complemented when the AI was tuned to a high true positive rate, because this complements the decision-makers’ own high true negative rate (Inkpen et al., 2022).

Complementarity may also be valuable when it comes to cognitive diversity (Clemen, 1989). Advice has been shown to be most valuable when the advisor contributes new information or a new thinking style. This is because judgments from those who are cognitively homogenous may err systematically (Rader et al., 2017). The idea of cognitive diversity encounters an interesting dilemma when it comes to advice from AI. AI is often viewed as complex (and “cognitively” different) to such an extent that human decision-makers are averse to using it. For example, many AI advisors do not provide advice in a way that is interpretable to humans (e.g., structured with features that are meaningful or understandable to the layperson; Rudin, 2019). Further, objective and analytical advice from AI may conflict with subjective and potentially intuitive cognitions from human decision-makers (Jarrahi, 2018). While maintaining a complementary degree of cognitive diversity, AI advisors should therefore be adjusted to suit the human mind (Burton et al., 2020), for example via algorithmic tuning to complement decision-makers’ strengths and weaknesses (Inkpen et al., 2022), or via discriminative and decision-theoretic modeling methods, as discussed in Wilder et al. (2020). This draws on the idea that human decision-making often involves intuitions and heuristics that contrast with the axioms of rational decision-making to which AI advisors are so closely tethered.

Importantly, although a focus on maximizing advice utilization via complementarity is a major avenue for future research, this should not be pursued without attention to potential problems. For instance, developers of AI should not wish to encourage blind overreliance on advice that is potentially incorrect (Gaube et al., 2023). Thus, complementary designs should seek to foster advice utilization while also providing decision-makers the opportunity to assess the decision processes and legitimacy of AI recommendations. For example, providing decision-makers with uncertainty estimates and/or confidence ratings can help reduce blind overreliance on AI advice (Bertrand et al., 2022).

#### 4.3.3 Environmental fit

Fit between the advising situation and either the AI advisor or the human decision-maker (or both) is also influential. Schneider and Freisinger (2022) specifically examined fit between a decision-maker’s task and procedure as an attempt to understand the mechanisms that influence algorithm aversion, and in an attempt to overcome individuals’ discounting of AI advice in situations that would benefit from utilizing it. Via a study on hospital triage decisions, the authors found that although the lack of emotions and rationality of AI advisors is helpful in medical decision contexts, there is a level of decision importance and accountability that makes doctors hesitant to use the advice blindly in such an environment.

Further research has speculated that a preference for AI over human expert advisors may be due to perceived fit between advisor and task characteristics (i.e., the capabilities of the AI meet the requirements of the task; Mesbah et al., 2021). In support of this theory, Hertz (2018) found that participants picked human advisors more for social tasks and AI advisors more for analytical tasks. This is echoed in the aforementioned research demonstrating decision makers’ preference for human advisors in situations that elicit affective (i.e., emotional) processing and AI advisors in situations that elicit utilitarian processing (Longoni and Cian, 2020; Larkin et al., 2022).

In summary, we contend that research on the impact of human-AI similarity and complementarity (i.e., fit-focused research) is part of an interactive research domain that more effectively serves to optimize collaboration between human decision-makers and AI advisors than previous, more static, research. The ability of AI to provide advice on complex decisions to a variety of individuals necessitates a more dynamic approach to the design of AI advisors, wherein AI can adapt its parameters to best suit the decision-maker with whom it is currently interacting, in the context of the decision at hand. This adaptation could occur either automatically (e.g., via machine learning) or at the behest of the human decision-maker (e.g., with the AI surveying decision-makers initially regarding their values and on an ongoing basis regarding their procedural preferences). By adopting a fit-focused lens, our review helps stakeholders to appropriately consider factors beyond merely the AI’s accuracy or technological advancement when approaching the selection of an AI advisor.

## 5 Discussion

This review examines the parallels and divergences between AI and human advice exchanges. As can be seen from the previous section of this review, we conclude that, although many insights can be extended from formative research on human advisors to the case of AI advisors, there are also considerable differences. Our review, however, also points to important areas for future research. In the current section, we discuss limitations of the current research that offer areas for future research, and we then discuss areas for future research that advance knowledge in ways other than addressing the limitations. We also advance several theoretical propositions across various topics. A summary of future research questions and theoretical propositions is provided in Table 4.

**Table 4:** Future research directions: research questions and associated theoretical propositions by topic area.

Topic Area	Research Question	Associated Theoretical Proposition(s)
Uniqueness	To what extent is advice from AI characterized as inherently “unique” (vis-à-vis the human decision-maker) and what influence does this have on advice solicitation?	The inherent uniqueness (due to different computational abilities) to human decision-makers of the information and recommendations provided by AI advisors, as compared to human advisors, makes AI advisors more attractive for novel tasks versus familiar tasks.
Multiple advisors	What is the impact of agreement (vs. disagreement) among multiple advisors (AI and human) on advice utilization and advisor evaluation?	Decision-makers will evaluate AI advisors more positively and weigh their advice more heavily when AI advice is more similar (vs. dissimilar) to human advice. Decision-makers will evaluate disagreement among advisors even more negatively when all the advisors are AI advisors than when all the advisors are human advisors. However, this negative reaction to disagreement among AI advisors can be ameliorated if the human decision-maker is made aware that the various AI advisors were trained on different information, use different algorithms, etc.—and if the various AI advisors are purposefully designed to “look” different (i.e., anthropomorphized in different ways).
Confidence	What is the impact of the conceptualization and display of AI advisor confidence on the decision-maker?	The relationship between AI advisor confidence (operationalized as probabilistic uncertainty or a confidence rating) and advice solicitation mimics the relationship between human advisor confidence and advice solicitation—that is, confidence increases advice solicitation—but the positive relationship is stronger for human decision-makers who display high numeracy and high comfort with technology (and in particular AI) than for those who do not.
Trust	How does decision-maker trust in an AI versus human advisor change over time?	Repeated interactions with an AI advisor will foster resilience to the trust breakdown that occurs following the presentation of inaccurate advice (in the context of an overall high level of AI advisor accuracy).
Social cost	What is the perceived social cost (e.g., to perceived competence; Lee, 2002) of AI advice as compared to human advice?	AI advice is perceived as less socially costly than human advice in terms of: (1) self-and other-perceptions of decision-maker competence, and (2) decision-maker embarrassment when asking for advice on sensitive topics. In some cases (e.g., skillful “prompting” of the AI advisor by the human decision-maker), AI advice may even bring social benefits that are not relevant to human advice.
Decision context	Can inconsistent findings on algorithm (i.e., AI) acceptance/appreciation versus aversion be reconciled by noting the specific conditions under which these studies were conducted?	The negative relationship between AI anthropomorphization and algorithm/AI aversion is moderated by task type, such that the relationship is weaker for analytical tasks than for social tasks.
Similarity	What is the impact of AI advisor and human decision-maker perceived similarity in terms of values, personality, and goals on advice solicitation and/or utilization?	AI advisor and human decision-maker similarity on values, personality, and goals will often increase advice solicitation and utilization. However, whether advice solicitation and utilization benefit more from similarity or complementarity will depend on the specific psychological construct in question. For instance, personality similarity will be more beneficial in the case of the personality trait of affiliation (or agreeableness) whereas personality complementarity will be more beneficial in the case of the personality trait of dominance (or autonomy; Tett and Murphy, 2002). Being aware of this, if given the ability to adjust the “personality” of an anthropomorphized AI advisor, human decision-makers will do so accordingly (e.g., decision-makers scoring high on affiliation will adjust AI advisor personality to also be high on affiliation whereas decision-makers scoring high on autonomy will adjust AI advisor personality to be low on dominance).
Complementarity	How are algorithmic acceptance/appreciation and advice utilization impacted by the extent to which the human decision-maker’s needs are met by the AI advisor?	The extent to which the human decision-maker’s needs regarding specific types of advice [e.g., receiving a specific recommendation regarding what to do or not to do; receiving a specific recommendation regarding what decision process to use or not to use; receiving information about decision options without an explicit recommendation; receiving social–emotional support; receiving an uncertainty estimate or a confidence rating; Dalal and Bonaccio (2010), Griffith et al. (2020), and Vodrahalli et al. (2022)] are met by the AI advisor is related positively to algorithm acceptance/appreciation and advice utilization.

The individual sections of the paper in which these theoretical propositions are discussed provide more detailed (and contextualized) explanations than can be provided in this table. Those sections of the paper also provide additional future research directions not listed in this table.

### 5.1 Limitations

Our review possesses some limitations that may help guide future research. One such limitation is that we used the literature on human advice as a “lens” through which to summarize research on AI advice. This approach is valuable because the human advice literature is more established than the AI advice literature, and because comparing findings on advice from humans to findings on advice from AI advisors has the potential to provide important insights. Further, this approach helps connect AI research to JDM research. However, it is possible that this perspective may have led us to neglect conclusions in the AI advice literature that have no analog in the human advice literature. Future research should explore this possibility.

A second limitation is that in our inclusion/exclusion criteria, we note that we did not focus on instances where AI itself makes decisions without human intervention (performative algorithms), instances where human input or advice facilitates a decision made by an AI (vs. human) decision maker, or instances in which a work team comprises some combination of human and AI members who must work together in a non-hierarchical decision-making team. We believe these exclusions are acceptable because we needed to maintain a reasonable scope for the review, and because these are relatively distinct phenomena–and ones that would not be as well informed by the human advice literature. However, these exclusions mean that we could not emphasize additional comparisons that may have been of interest to some readers–for example, how findings differ across the case of AI advisors and human decision makers versus the case of human advisors and AI decision makers.

A third limitation is that the current manuscript does not specifically draw conclusions regarding the relative importance of the identified characteristics (e.g., confidence, trustworthiness) for human and AI advice interactions. This decision was made because there does not yet exist sufficient primary research to support such conclusions; however, future research should seek to establish the relative importance of these focal characteristics in the context of advice exchanges for humans and for AI.

A final limitation is that chatbots such as ChatGPT are used not only for advice but also for material help, such as writing software code. This type of material help is not within the scope of the current review because the oversight provided by the human decision maker differs across material help versus advice: for instance, checking code provided by a chatbot is qualitatively different from agreeing or disagreeing with a recommendation from a chatbot. However, future research should review the literature on the provision of material help from a chatbot.

### 5.2 Future research directions

Below, we discuss areas for future research that advance knowledge in ways other than addressing the limitations of the current study.

#### 5.2.1 Uniqueness

An overarching area for future research stems from themes in the human advice literature for which corresponding research using AI advisors is scarce or nonexistent. One such theme is the impact of the provision of unique information by an advisor—that is, information not already possessed by the decision-maker (or other advisors, if any). Van Swol and Ludutsky (2007) demonstrated that the provision of unique information increases subsequent advice solicitation from human advisors, and Hütter and Ache (2016) found that the provision of advice dissimilar to the decision-maker’s original opinion increased advice solicitation. Future research should determine if this relationship is analogous for AI advisors. For instance, might information from AI advisors be perceived as unique or dissimilar simply due to its origin (i.e., coming from AI vs. a human)? Additionally, a large stream of research has been dedicated to the modeling of human intuitive processing and information processing, with one underlying goal being to align human and AI decision processing (Burton et al., 2020). The aforementioned findings from human advice research, however, perhaps suggest that some discrepancies between human and AI information processing and decision-making styles may foster advice solicitation. More research is therefore needed to determine the extent to which advice from AI is characterized as inherently “unique,” and the influence this has on advice solicitation and utilization.

#### 5.2.2 Multiple advisors

An additional theme concerns the influence of multiple advisors on advice utilization. Research on AI advice has not sufficiently examined the impact of agreement (vs. disagreement) amongst multiple advisors (AI and human) on advice utilization. Research on human advice has supported the idea that decision-makers make deductions about the accuracy and expertise of multiple advisors by assessing their level of agreement (Budescu and Yu, 2007; Kämmer et al., 2023). Specifically, decision-makers place less weight on advice, and utilize advice less, when the estimates from multiple advisors are discrepant (Kämmer et al., 2023). A somewhat comparable vein of research in AI advice is that on hybrid forecasting, which examines how human and AI forecasts—or more broadly judgments—can be combined to produce judgments more optimal than either human or AI judgments independently. An important facet of this research involves exploring the contexts in which decision-makers will be more amenable to hybrid advice (i.e., advice that combines human and AI sources; Himmelstein and Budescu, 2023). For example, future research should examine if decision-makers evaluate advisors more positively and are more willing to utilize hybrid advice when advice from the human and AI advisors does not conflict.

An additional area for future research involves human decision-maker reactions to multiple AI advisors that provide conflicting advice. The tendency to discount conflicting advice from multiple human advisors (Kämmer et al., 2023) may be exacerbated in the case of conflicting advice from multiple AI advisors because humans may perceive all forms of AI to be similar to each other, and may therefore find discrepancies among AI advisors to be particularly inexplicable and problematic. It is possible that this adverse reaction could be ameliorated if the human decision-maker is made aware that the various AI advisors were trained on different sources of information, use different algorithms, and so forth—and if the various AI advisors are purposefully designed to “look” different (e.g., different appearances, voices, and “personalities” if the AI advisors are anthropomorphized).

#### 5.2.3 Debiasing interventions

Cognitive biases (the application of heuristics to environments for which they are ill-suited; Gigerenzer and Brighton, 2011; Kliegr et al., 2021) can impact human-AI interactions in several ways. For example, pre-existing cognitive biases can influence how decision makers evaluate and utilize AI, and AI systems can also provoke or amplify decision-makers’ cognitive biases (Bertrand et al., 2022). In general, findings on solicitation and utilization of advice from AI suggest that human decision-makers’ preference for advice (i.e., human vs. AI advice) is not always completely rational or optimal. Accordingly, cognitive schemas can lead decision-makers to seek human advice over AI advice when they have seen the AI advisor err, even if the AI advisor typically outperforms the human advisor (Dietvorst et al., 2015; Reich et al., 2022).

Regarding the amplification of existing biases, AI systems can trigger biases such as recognition bias, causality bias, framing bias, etc. (Bertrand et al., 2022). For example, an AI advisor designed to cater to decision maker preferences may lead to confirmation bias, such that the decision maker’s preferences become an informational echo chamber. Research on advice from AI would thus do well to draw on the human advice literature that has examined the effectiveness of debiasing interventions on increasing utilization of advice (Yoon et al., 2021). For example, Yoon et al. (2021) found that administering an observational learning-based training intervention to participants could reduce cognitive biases and lead to greater advice taking. However, it should be noted that the JDM literature suggests that debiasing is very difficult and that most interventions are unsuccessful. Future research can seek to develop and test the effectiveness of learning-based training interventions that focus on reducing AI-specific cognitive biases or schemas (e.g., the aforementioned perfection schema) with the goal of increasing AI advice utilization. For example, these interventions could help demonstrate that AI decision-making processes can be adaptable, and that AI mistakes can be detected and corrected in a way similar to (or better than) humans. In support of this idea, research has shown that demonstrating an AI advisor’s ability to learn can reduce reluctance in their advice (Berger et al., 2020). Research should also continue to build on techniques to mitigate cognitive biases by exploring different contexts in which certain biases might occur (e.g., various environments and task types; Bertrand et al., 2022).

#### 5.2.4 Operationalization of advice utilization

Research on advice from humans has suggested that substantive findings may be impacted by the way in which advice utilization is operationalized (Bonaccio and Dalal, 2006; Dalal and Baines, 2023). Operationalizations include matching (i.e., the match between the advisor’s recommendation and the decision-maker’s choice), “weight of advice” (an assessment of how much the decision-maker moves toward the advice), and, less commonly, multiple-regression-based approaches. Advice utilization is also often measured using self-report measures of advice utilization or even advice utilization intention (Van Swol et al., 2019). The extent to which different operationalizations yield convergent findings is unclear even in the human advice literature (Dalal and Baines, 2023), let alone in the AI advice literature or the literature comparing human and AI advice. This is an important barrier to meta-analytic cumulation of results. What is therefore needed is research involving a series of decisions, across different domains (e.g., financial, ethical, and aesthetic) and procedural variations, and involving either human or AI advice (or both), with the aim of determining the extent to which various formula-based, regression-based, and self-report operationalizations of advice utilization yield convergent findings as well as the contextual factors that affect the extent of their convergence (Dalal and Baines, 2023).

#### 5.2.5 Confidence

Future research should additionally determine how AI advisor confidence is most effectively *conceptualized*, and how it is most effectively *displayed* to the human decision-maker (e.g., as a range akin to a confidence interval vs. as a rating on a scale from low to high confidence). This research should compare the influence of AI versus human advisor confidence on decision-makers, both overall and across various ways of conceptualizing and displaying confidence. It is possible that the strength of the positive relationship between advisor confidence and human decision-maker advice solicitation from the advisor is similar regardless of whether the advisor is human or AI. Alternatively, it is possible that this is only true for decision-makers scoring high in numeracy and prior experience/comfort with AI, whereas decision-makers scoring low on these constructs would simply exhibit low advice solicitation from AI advisors across the board and therefore (i.e., due to this range restriction), exhibit a weaker positive relationship between advisor confidence and decision-maker advice solicitation from the advisor. Future research should explore questions such as these.

#### 5.2.6 Social cost and benefit

Another theme in the human advice literature reveals that decision-makers’ fear of appearing incompetent hinders advice solicitation (Brooks et al., 2015; MacGeorge and Van Swol, 2018; Lim et al., 2020). However, research has found that, rather than diminishing perceptions of competence, advice-seeking can, at least under some circumstances, elevate others’ perceptions of the advice-seeker’s competence (Brooks et al., 2015; Palmeira and Romero Lopez, 2023). Yet, even when others perceive them to be more competent because they have sought advice, people may often perceive themselves as less competent as a result of having done so (Brooks et al., 2015). Social costs such as reputational and face costs (Lee, 2002; MacGeorge and Van Swol, 2018) may, however, be lower for AI advice than human advice because obtaining advice from AI can have a higher level of anonymity than obtaining advice from another individual, and is additionally becoming increasingly normalized for the most trivial of tasks.

AI advisors may additionally be preferred to their human counterparts with regard to another social cost: embarrassment. When seeking advice on sensitive topics (e.g., medical conditions of a sexual nature, crimes committed, or embarrassing mistakes made at work), decision-makers may believe that advice from AI advisors is anonymous and free of social judgment, and may therefore prefer AI advisors to human advisors (Pickard et al., 2016; Branley-Bell et al., 2023). Interestingly, however, some research suggests that findings may not be as cut-and-dried, and that the benefits of anonymity may be masked by factors such as the perceived warmth/likability and domain-specific competence of the AI versus human advisor (Hsu et al., 2021). Perhaps anthropomorphized AI advisors would represent the best of all worlds in the sense of being seen as experts (e.g., by displaying an avatar wearing a white coat and stethoscope, signifying medical expertise) and likable (e.g., by smiling and exhibiting enthusiasm) yet simultaneously anonymous (by virtue of being an AI rather than human advisor; Hsu et al., 2021).

Interestingly, obtaining advice from AI may also have the potential to accrue social benefits that have no parallel when obtaining advice from humans. For instance, the human decision-maker may impress others by exhibiting considerable skill in the use of “prompts” to an AI advisor, thereby obtaining higher-quality advice than others would have been able to obtain from the same AI advisor in a given situation. In this case, seeking advice from AI publicly (vs. anonymously) may be beneficial. Future research should therefore examine the conditions under which AI advice reduces social costs and increases social benefits, the role played by anonymity, and the factors that may mask (e.g., interact statistically with) the role of anonymity.

#### 5.2.7 Decision context

An additional overarching area for future research concerns the areas of research in which findings have been inconsistent. Largely, these inconsistencies exist in research on acceptance versus discounting of AI advice. For instance, although there is significant evidence that human decision-makers are averse to AI advice (Dietvorst et al., 2015; Castelo et al., 2019; Burton et al., 2020; Jussupow et al., 2020), research is increasingly revealing the absence of aversion to AI advice (Ben-David and Sade, 2021) or even appreciation for AI advice (Logg et al., 2019). We suggest that these inconsistencies can largely be reconciled by noting the specific conditions under which these studies were conducted.

For instance, research has begun to reveal that decision-makers might experience algorithm aversion on tasks deemed to be subjective versus algorithm appreciation on tasks deemed to be objective (Castelo et al., 2019). An additional decision context that remains to be examined, however, is the extent to which the timing of advice impacts decision-maker reactions to advice. Some research in the human advice literature (e.g., Sniezek and Buckley, 1995; Schrah et al., 2006) has examined this issue, finding that decision makers sometimes choose to access advice in a confirmatory sense, after having already conducted their own information search and reached an initial opinion. In a study on AI advice, Wise (2000) noted that decision-makers received advice after having generated a solution themselves, and that outcomes may have been different if the advice were presented earlier in the decision-making process. More research should therefore be conducted to examine the impact of timing of AI advice on decision outcomes.

#### 5.2.8 Fit

Research should more carefully note the characteristics of the advisor, decision-maker, and environment that may be impacting the advice exchange and its outcomes. The model put forth in the current paper (see Figure 1) is intended to be a helpful means toward that end. Future research should also compare the relative importance of the three aspects of fit discussed in the model, namely: (1) fit between AI advisor characteristics and human decision-maker characteristics, (2) fit between AI advisor characteristics and environmental characteristics, and (3) fit between human decision-maker characteristics and environmental characteristics. As noted previously, fit can be conceptualized in terms of similarity or complementarity.

##### 5.2.8.1 Similarity

In the research literature in organizational psychology/behavior, fit based on similarity is referred to as “supplementary fit” (Edwards, 2008). Applied to the current case, the idea is that the AI advisor can supplement or enhance the human decision-maker by virtue of similarity between the two (cf. Tett and Murphy, 2002; Edwards, 2008). To examine the role of similarity, future research should assess whether decision-makers’ extent of perceived value similarity, personality similarity, and/or goal similarity with AI advisors (or their human or organizational developers and providers) influences advice solicitation and/or utilization. Regarding personality similarity, not all AI advisors currently display, or would benefit from displaying, what could be considered “personality” traits; however, personality similarity may be important for certain AI advisors such as chatbots or other conversational agents such as social robots (Ta et al., 2020).

A question for future research related to this point is: when decision-makers are able to stipulate the “personality” of their AI advisor, will they choose a personality similar to what they perceive to be their own personality? One possibility is that decision-makers will stipulate levels of personality traits in AI advisors that provide themselves (i.e., the decision-makers) opportunities for personality trait expression. For some personality traits, this may indeed take the form of personality similarity: for instance, decision-makers who score high on affiliation (or agreeableness) may be more likely than most to prefer advisors who also score high on affiliation (Tett and Murphy, 2002). For other personality traits, however, this may take the form of personality complementarity: for instance, decision-makers who score high on autonomy may be more likely than most to prefer advisors who score low on dominance (Tett and Murphy, 2002). We discuss complementarity further in the next subsection.

Goal similarity is also likely an important aspect of fit between a decision-maker and AI advisor. For instance, AI developers may focus on maximizing computational fairness criteria (e.g., via disparate impact testing or adversarial debiasing; Linardatos et al., 2020), whereas decision-makers, who are often organizational stakeholders, may wish to emphasize procedural and distributive justice criteria (Köchling and Wehner, 2020). Examples of such justice-related criteria include neutrality, consistency, and correctability (among many others) for procedural justice and specific allocation rules (e.g., equity or equality or need) for distributive justice (Colquitt, 2001). Thus, the goals of the AI advisor should be made salient via the developer and provider of the AI advisor, such that the decision-maker can determine if goal similarity exists.

##### 5.2.8.2 Complementarity

In the research literature in organizational psychology/behavior, fit based on complementarity is referred to, perhaps unsurprisingly, as “complementary fit” (Edwards, 2008). Applied to the current case, the idea is that the strengths of the AI advisor can complement or offset the weaknesses of the human decision-maker (*cf.* Tett and Murphy, 2002; Edwards, 2008). To examine complementarity, future research should evaluate the decision-maker’s “need fulfillment” by the AI advisor (*cf.* Tett and Murphy, 2002). In the previous subsection, we discussed how personality fit between the AI advisor and human decision-maker may sometimes take the form of complementarity instead of similarity. However, several other examples of complementarity, in the form of need fulfillment, may also be relevant.

For instance, the impact of the specific type(s) of advice provided by the advisor is greatly understudied in the human advice literature (Bonaccio and Dalal, 2006; Dalal and Bonaccio, 2010), let alone in the AI advice literature. After all, an advisor may offer numerous types of advice individually or in some temporal combination: for instance, a specific recommendation regarding what to do or what not to do, a recommendation about the decision process to use or not to use, information about decision options without an explicit recommendation, social–emotional support, and/or an expression of confidence or uncertainty (Dalal and Bonaccio, 2010; Griffith et al., 2020; Vodrahalli et al., 2022). It seems reasonable to posit that “algorithm appreciation” (and subsequent advice utilization) stems from the extent to which the decision-maker’s needs regarding specific types of advice are met by the AI advisor. If so, this suggests that AI advisors should be designed such that they can be tuned by human decision-makers as per their needs. See Table 4 for theoretical propositions.

An additional aspect of human-AI complementarity is the role of interactions between various characteristics of the AI advisor, or the interactions between various characteristics of the human decision maker. Specifically, compared to between-entity interactions (e.g., interactions between a characteristic of the AI advisor and a characteristic of the human decision maker, such as in the case of personality fit), within-entity interactions (e.g., interactions between several characteristics of the AI advisor) may have a further impact on the decision-maker and/or the advisor. Consider, for example, that previous human advice research has focused on the joint effect of advisor expertise and confidence on advice utilization by decision-makers (Bonaccio and Dalal, 2010). In other words, decision-makers’ needs in terms of uncertainty reduction (Lim et al., 2020) are seemingly fulfilled by the juxtaposition of advisor expertise and advisor confidence in their recommendations to an appreciably greater extent than by advisor expertise alone or advisor confidence alone. Therefore, future research should examine how the interactive effects of AI advisor characteristics function to impact decision-maker reactions to advice. For example, future research could use a policy capturing design (Aiman-Smith et al., 2002; Zhu et al., 2022) to simultaneously determine if numerous AI advisor characteristics (expertise and confidence, expertise and transparency, expertise and affiliation in the case of anthropomorphized AI advisors, etc.) interact synergistically to aid decision-maker advice seeking and/or utilization.

#### 5.2.9 Stability of findings

A separate area for future research involves seeking to determine the relative stability of the aforementioned findings, given rapid improvements in AI capabilities. Although many human individual differences are likely to maintain consistency or at best small changes over time, human familiarity and comfort with AI are likely to change, and more specifically increase, rapidly over time. Thus, effects like algorithm aversion and theories like the uncanny valley (Gray and Wegner, 2012; Jussupow et al., 2020; Lucien, 2021; Mahmud et al., 2022) may receive less support in future years. Research should expend effort toward modeling the hypothesized direction of social and affective responses to AI advice with regard to developments in technology.

## 6 Practical implications

The practical implications of this review are manifold. This research has implications for the development of practice and policy regulations regarding the use of AI in organizational decision-making. Given decision makers’ preference for human advisors in situations that elicit affective processing, and AI advisors in situations that elicit utilitarian processing (Hertz, 2018; Longoni and Cian, 2020; Larkin et al., 2022), at first thought it may seem as though AI advisors in organizations should be implemented for objective tasks or tasks with high computational needs, but not for more subjective tasks or tasks with heavy social and/or emotional content. However, as pointed out by Castelo et al. (2019), for those subjective tasks (e.g., making a numerical estimate) or social–emotional tasks (e.g., rating the attractiveness of an individual) that would nonetheless benefit from the use of an algorithm, increasing the anthropomorphization of an AI advisor could be an effective way to increase AI usage. Further, framing effects (e.g., emphasizing the competence of the AI advisor, or framing the task as benefitting from quantitative rather than intuitive analysis; Castelo et al., 2019; Hou and Jung, 2021) could increase AI advice utilization in certain contexts. Finally, AI is rapidly improving in its ability to detect and analyze emotions (Nandwani and Verma, 2021; Joshi and Kanoongo, 2022), indicating that the utilitarian versus affective decision distinction may soon carry less weight in decision-makers’ preference for an AI versus a human advisor.

Beyond pursuing “person”-environment fit between the AI advisor and the task, organizations can also pursue “person”-person fit between the AI advisor and the human decision-maker. Findings that higher decision-maker numeracy correlates with greater acceptance of AI advice (Logg et al., 2019; Willford, 2021) suggest that organizations should make extra efforts to facilitate the use of AI advice by decision-makers with lower numeracy, given that these may be the decision-makers likely to benefit most from AI advice.

Another recommendation for practice involves the facilitation of employee trust in AI. Organizations and developers can facilitate trust in AI advice by increasing transparency and explainability, and by prioritizing (and making salient to decision-makers) technical robustness and bias minimization as well as privacy and data governance (Walsh et al., 2019; Bianchi and Briere, 2021). Trust in AI advice may also be fostered by factors such as perceived similarity of the AI advisor to humans, or sensitivity on the part of the AI advisor to socio-emotional states of the human decision-maker (Hertz, 2018). Given that anthropomorphization may increase trust in AI advisors (Pak et al., 2012) and may lead to increased advice utilization, organizations and developers may wish to intentionally implement AI advisors with human features and characteristics. However, efforts to increase transparency should be made with the caveat that transparency may be less effective for simple AI than for complex AI, given that human decision makers’ high expectations of AI may mean that the utility of simple AI may be erroneously underestimated (Lehmann et al., 2022). Therefore, perhaps simple (vs. complex) transparent AI advisors should be accompanied by an explanation of or testament to their effectiveness, in an effort to avoid misplaced underutilization due to simplicity.

Relatedly, organizations wishing to implement AI advisors should be sure to assess, and attempt to minimize, technology-related anxiety on the part of their human employees. This can be accomplished through training programs aimed at increasing competence with using technology and interacting with AI advisors in particular (Lindblom et al., 2012). However, it can also be accomplished through the design of AI interfaces that are intuitive and non-technical for human users, including those who are relatively unfamiliar with and averse to technology.

Organizations should also be aware of the potential repercussions of erroneous AI advice (Madhavan and Wiegmann, 2007). Given the idea that AI mistakes tend to be weighted more heavily than human mistakes, organizations should create contingency plans to mitigate decision-maker concerns about AI efficacy. These contingency plans can be aimed at reducing unhelpful biases and response tendencies on the part of human decision-makers (Madhavan and Wiegmann, 2007). For example, organizations can provide reminders concerning AI’s accuracy, both in an absolute sense and relative to that of comparable humans.

Given the considerable ethical and legal considerations surrounding the use of AI for providing advice in organizations (e.g., the parity problem), the increasing adoption of AI advice also has important practical implications for human resource (HR) management (Köchling and Wehner, 2020; Langer et al., 2020; Pena et al., 2020; Hunkenschroer and Luetge, 2022). First, AI advice is likely to have a large influence on HR practices such as employee recruitment and personnel selection. The impact (positive and negative) of AI-based recruitment tools has already begun to receive the spotlight: for instance, the British multinational consumer goods company Unilever has been open about its use of AI to (seemingly successfully) recruit new employees (Marr, 2019). Research has suggested that the use of AI can make employee selection more systematic by reducing bias against groups of employees who are already underrepresented in various employment settings (Lepri et al., 2018; Sajjadiani et al., 2019). However, this is not always the case: it is by now well-known that AI can itself display biases if its input data are biased or unrepresentative, and that AI may in some cases even amplify human biases (Chander, 2017; Köchling and Wehner, 2020; Mehrabi et al., 2021). Bias in AI systems can also arise as a function of their design [e.g., due to flawed selection of criterion, predictor set, and algorithm; Landers and Behrend (2023)], rather than due solely to biased input data [e.g., if there is range restriction; Mehrabi et al. (2021) and Landers and Behrend (2023)].

Thus, AI advice used in an employee recruitment and selection context should be expected to meet the same quality standards required of more traditional recruitment and selection tools (Nye et al., 2023). For example, AI recruitment or selection advice should have a clear relation to relevant job performance outcomes, should provide validity evidence (e.g., convergent, discriminant, and criterion-related validity), should be fair and unbiased, and should be implemented with specific organizational needs in mind (Nye et al., 2023).

There are also practical implications concerning the impact of AI advice on employee development and performance management systems in organizations (Köchling and Wehner, 2020). Organizations have begun to use recommender systems to evaluate and promote employees (e.g., IBM Watson Talent Career Coach for Career Management, n.d.; Köchling and Wehner, 2020). Despite the purported benefits of these systems, organizations and individual stakeholders must be aware of the potential pitfalls of implementing AI advisors (Köchling and Wehner, 2020). In terms of helping employees develop skills, knowledge, and abilities, AI is immensely beneficial in predicting variables of interest to the HR department and collecting data from employees (Köchling and Wehner, 2020). A benefit to AI *advice*, as opposed to AI decision-making, is that final decisions are made by humans, rather than AI (Köchling and Wehner, 2020). This may increase employees’ perceptions of the validity and fairness of internal HR processes (Kaibel et al., 2019). Therefore, we recommend that organizations strategically select which decisions should be made by humans (with advice from AI) versus by more autonomous/unsupervised AI.

## 7 Conclusion

The current review integrates existing research on advice from humans with advice from AI. Prompted by inconsistencies in organizational scholars’ understanding of how AI alters individuals’ gathering and usage of evidence for decision making, we put forth a conceptual framework that incorporates advisor and advisee characteristics, advice/decision characteristics, and advice outcomes–and we present our findings within this framework. We encourage future research to examine AI advice exchanges in a context that acknowledges the dynamic nature of the advice exchange process and assesses the relative contributions of individual differences and environmental/task characteristics in advice exchanges and outcomes.

## Author contributions

JB: Conceptualization, Investigation, Methodology, Supervision, Writing – original draft, Writing – review & editing. RD: Conceptualization, Methodology, Supervision, Writing – original draft, Writing – review & editing. LP: Investigation, Writing – original draft, Writing – review & editing. H-CT: Investigation, Writing – original draft, Writing – review & editing.

## Footnotes

^1^Traditional search engine search results are not considered AI advice because they were not designed to extend advice in the context of a decision; rather, traditional search engines served to organize information and make it accessible to users (Google, n.d.). However, newer AI-enabled chatbot search via Bing, Google, etc., could be considered an AI advisor, given that part of its purpose is to offer tailored advice to queries or prompts (Kachalova, 2023).

^2^Knowledge gained by the decision-maker via advice during the decision process is an outcome in our model; conversely, knowledge held by the advisor prior to the decision is part of what constitutes the advisor’s expertise. It is the latter to which we refer here.
